# Calcofluor White Combination Antifungal Treatments for *Trichophyton rubrum* and *Candida albicans*

**DOI:** 10.1371/journal.pone.0039405

**Published:** 2012-07-06

**Authors:** Joanne M. Kingsbury, Joseph Heitman, Sheldon R. Pinnell

**Affiliations:** 1 Department of Molecular Genetics and Microbiology, Duke University Medical Center, Durham, North Carolina, United States of America; 2 Department of Dermatology, Duke University Medical Center, Durham, North Carolina, United States of America; 3 Skin Science Institute Inc., Durham, North Carolina, United States of America; Louisiana State University, United States of America

## Abstract

Superficial mycoses caused by dermatophyte fungi are among the most common infections worldwide, yet treatment is restricted by limited effective drugs available, drug toxicity, and emergence of drug resistance. The stilbene fluorescent brightener calcofluor white (CFW) inhibits fungi by binding chitin in the cell wall, disrupting cell wall integrity, and thus entails a different mechanism of inhibition than currently available antifungal drugs. To identify novel therapeutic options for the treatment of skin infections, we compared the sensitivity of representative strains of the dermatophyte *Trichophyton rubrum* and *Candida albicans* to CFW and a panel of fluorescent brighteners and phytoalexin compounds. We identified the structurally related stilbene fluorescent brighteners 71, 85, 113 and 134 as fungicidal to both *T. rubrum* and *C. albicans* to a similar degree as CFW, and the stilbene phytoalexins pinosylvan monomethyl ether and pterostilbene inhibited to a lesser degree, allowing us to develop a structure-activity relationship for fungal inhibition. Given the abilities of CFW to absorb UV_365 nm_ and bind specifically to fungal cell walls, we tested whether CFW combined with UV_365 nm_ irradiation would be synergistic to fungi and provide a novel photodynamic treatment option. However, while both treatments individually were cytocidal, UV_365 nm_ irradiation reduced sensitivity to CFW, which we attribute to CFW photoinactivation. We also tested combination treatments of CFW with other fungal inhibitors and identified synergistic interactions between CFW and some ergosterol biosynthesis inhibitors in *C. albicans*. Therefore, our studies identify novel fungal inhibitors and drug interactions, offering promise for combination topical treatment regimes for superficial mycoses.

## Introduction

Dermatophytoses, the infections of keratinized tissues such as the skin, hair and nails by the highly specialized dermatophyte fungi such as *Trichophyton rubrum,* represent the most common type of human infection worldwide, particularly in aging, diabetic or immunocompromised individuals [1], [2], [3], [4], [5]. Current therapeutic options for treatment of superficial mycoses rely on topical or oral applications of drugs including griseofulvin (targets microtubules), polyenes (amphotericin B and nystatin and natamycin), terbinafine (allylamine), miconazole (imidazole) and other azole drugs such as itraconazole (all of which target ergosterol biosynthesis/membranes) [3], [6], [7], [8]. However, treatment efficacy is limited by drug side effects including hepatotoxicity, narrow spectrum of action, long duration of treatment, cost and the development of microbial drug resistance [3], [6], [9]. Therefore, the identification of new antifungal drugs and treatment regimes is necessary. One method to improve treatment efficacy is by antifungal combination regimes as certain combination treatments exhibit synergistic antifungal action, as well as providing broader spectrum of activity, and reducing the chance of drug resistance arising. Given the accessibility of skin infections to light, photodynamic therapy whereby selective killing from oxidative damage is mediated by a combination of sensitizing drug and photons of light of a wavelength specific to the sensitizer’s absorption properties, offers an alternative therapeutic approach for dermatophytoses. Specifically, a number of sensitizers, particularly porphyrin compounds such 5,10,15-tris(4-methylpyridinium)-20-phenyl-[21H,23H]-porphine trichloride (Sylsens B) and the porphyrin precurser 5-aminolevulinc acid, combined with light at various wavelengths and sources, have been found to be effective against *T. rubrum* and other fungi both *in vitro* and *in vivo* (reviewed in [10]).

Fluorescent brighteners are typically diaminostilbene derivative compounds that fluoresce upon exposure to UV light, and bind through hydrogen bonding to β-linked fibrillar polymers such as cellulose and chitin [11]. Due to these properties, fluorescent brighteners such as calcofluor white (CFW) have been used extensively in the textile, detergent and paper industry for creating a whitening effect, as well as in fungal diagnostics and research [12], [13], [14], [15]. In fungi, binding of fluorescent brighteners to nascent chitin chains affects normal chitin assembly by competing for hydrogen bonding sites, and because chitin is an essential component of fungal cell walls, fluorescent brightener binding compromises cell wall integrity, inhibiting fungal growth [16], [17], [18], [19], [20], [21]. Even though chitin comprises the innermost of three layers in cell walls of dermatophytes such as *T. rubrum* (outer layer β–glucans, second layer galactomannan, inner layer chitin), differing from those of yeast such as *C. albicans* (outer layer mannoprotein, inner layers β–glucans and chitin), the staining pattern for fluorescent brighteners 220 and 119 indicates that binding predominantly occurs at the chitin layer, causing substantial perturbation of the entire cell wall layer ultrastructure [17]. Fluorescent brighteners are considered nontoxic to mammals, verified by comprehensive toxicology studies performed due to the extensive use of these products in the textile industry (eg [22], [23]). Therefore, fluorescent brighteners may have utility as topical drug treatments.

To identify novel dermatologic therapeutic options, we evaluated the inhibition of two divergent, dermatologically-relevant species *T. rubrum* (the most commonly isolated fungus from dermatophyte infections [3], [24], [25]), and *Candida albicans* (the most significant yeast contributor to skin infections [3], [4], [26]) by a panel of stilbene and non-stilbene fluorescent brighteners, and determined a structure-activity relationship for drug efficacy. Given the ability of CFW to selectively bind fungi and absorb light at the wavelength 365 nm, we explored the antifungal effects of combination CFW/UV_365 nm_ irradiation treatment. We also determined drug interactions between CFW and recognized fungal inhibitors for topical drug application.

## Materials and Methods

### Strains, Media and Growth Conditions

Strains used in this study included the sequenced reference strain *C. albicans* SC5314 [27] and the CLSI-recommended reference strain *T. rubrum* ATCC MYA-4438 [28], [29], as well as *T. rubrum* MR851, MR1505, MR1461, and MR827 (provided by Dr. R. Barton, University of Leeds, UK). *C. albicans* was cultured on Yeast Peptone Dextrose (YPD) plates or broth, and *T. rubrum* was cultured on Potato Dextrose Agar (PDA). For MIC assays for both species, RPMI 1640 (Sigma R1383) was supplemented with dextrose (2 g/L) and MOPS (34.54 g/L), and the pH was adjusted to 7.0. For disc diffusion assays, RPMI 1640 medium was solidified with agar (20 g/L). Plates were incubated at 30°C for 1 or 2 days (*C. albicans*) or 5 days (*T. rubrum*), or at room temperature for least 14 days for *T. rubrum* conidiation.

### Drug Preparation

Drugs were dissolved in DMSO, ethanol, 1 M NaOH, or water, and used at the working concentration ranges indicated in Table S1. For testing drug interactions, concentration ranges of CFW included 0.179–11.46 µg/ml (for *T. rubrum*) and 0.72–45.85 µg/ml (for *C. albicans*), 0.19–12.0 µg/ml of fluorescent brightener 113, and 0.34–21.82 µg/ml of fluorescent brightener 85. Ten-fold concentrations of drugs were serially diluted two-fold, and 20 µl aliquots were added to the wells of 96-well flat-bottomed microtitre plates. When the drug solvent comprised DMSO, ethanol or 1 M NaOH, the diluent consisted of 10% (vol/vol) DMSO or ethanol or 0.1 M NaOH, respectively, thus the final concentration of DMSO or ethanol in assays was 1% (vol/vol), or 0.01 M NaOH. When testing drug interactions, 10 µl of 20-fold dilutions of each drug were added to assay wells.

### MIC and MFC Assays

Minimum inhibitory concentration assays (MIC) were prepared for *T. rubrum* ATCC MYA-4438 and *C. albicans* SC5314 as described previously [29], [30]. Briefly, the *C. albicans* SC5314 inoculum was prepared from an overnight YPD culture that had been washed twice in sterile dH_2_0, and resuspended in RPMI medium to a concentration of 1.1×10^3^ cells/ml, determined using a hemocytometer. The *T. rubrum* ATCC MYA-4438 inoculum was prepared to a concentration of 1.1×10^4^ microconidia/ml by washing microconidia off a PDA plate culture with 5 ml sterile dH_2_0, filtering through an 8 µm Whatman filter, and washing the filtrate with dH_2_0. An aliquot of inoculum was diluted accordingly and plated to agar plates in triplicate to determine the exact viable cell concentration. Volumes of 180 µl of cells were added to the 20 µl drug dilutions in 96-well microtitre plate wells. Plates were sealed and following incubated for 5 days (*T. rubrum*), 24 h (*C. albicans* MICs for clotrimazole, voriconazole, thioconazole, and itraconazole combinations), or 48 h (all other *C. albicans* MICs). The shorter 24 h incubation time for *C. albicans* with various azoles was used to circumvent the confounding trailing effect observed following 48 h incubation with these drugs, as recommended previously [31], [32], [33]. Since drugs were tested for their potential as topical treatments, microtitre plates were incubated at the physiologically relevant temperature of 30°C (while the average skin temperature is affected by clothes coverage, location, air temperature, or patient age, the mean temperature of uncovered human skin at room temperature (23–25°C) is 31.5°C, with foot temperature (site of onychomycosis) even lower [34], [35], [36]). 30°C is also the temperature recommended by American Type Culture Collection for the growth of both *C. albicans* SC5314 and *T. rubrum* ATCC MYA-4438 (www.atcc.org), and commonly for these species in antifungal susceptibility studies, (for example [37], [38], [39], [40], [41], [42]). Following incubation, the optical density at wavelength 600 nm (OD_600_) was read using a Tecan Sunrise plate absorbance reader. The MIC_80_ was designated the lowest concentration of drug/treatment condition resulting in at least 80% reduced growth compared with the OD_600_ reading of the no-drug/treatment control.

To determine minimum fungicidal concentrations (MFC), the entire contents of wells containing no visible growth were plated to YPD (*C. albicans*) or PDA (*T. rubrum*). The MFC was defined as the lowest concentration of drug/treatment resulting in an at least 99% (*T. rubrum*, MFC_99_) reduction of viable cfu from the inoculum. For *C. albicans*, the 10-fold lower inoculum size precluded the determination of 99% killing with a high level of accuracy; thus a 95% reduction (MFC_95_) endpoint was employed. All MIC and MFC experiments were performed in triplicate.

The Fractional Inhibitory Concentration (FIC) was employed to quantify drug interactions and was calculated as follows: FIC  =  [(MIC_80_ of drug A in combination/MIC_80_ of drug A alone)/(MIC_80_ of drug B in combination/MIC_80_ of drug B alone)]. In accordance with ASM guidelines, interactions with FIC ≤0.5 were considered synergistic, indifferent if >0.5 to ≤4.0, and antagonistic if >4.0 [43].

### UV_365 nM_ Treatment

To test the effect of irradiation on cell inhibition by CFW, microtitre plates containing CFW, RPMI media and *C. albicans* or *T. rubrum* were set up as described for MIC assays, and wells were individually irradiated at a peak wavelength of 365 nm using a Hamamatsu LC-L2 UV-LED module with 12 mm wide diode (LED head unit L10561 series, control box model C10608, driver hub unit model C10558). The diode was held directly over wells so that the edge of the diode touched the top of the microtitre plate, ensuring the diode was equidistant from cultures in each experiment (the irradiation distance from the light source to the well bottom was approximately 15 mm). To circumvent light exposure to neighbouring wells during irradiation, black-walled assay 96 well plates (Corning Costar) were used, and wells were covered before and immediately after irradiation with foil. Light intensity was measured using a light power meter (Model C6080-13, Hamamatsu Photonics K.K.), equipped with a 365 nm detector (model FD1591) and averaged 624 mW/cm^2^ when the distance from the light source was 5 mm. Wells were irradiated for 0, 30 (∼18.7 J/cm^2^), 60 (∼37.4 J/cm^2^), 90 (56.2 J/cm^2^) or 120 seconds (74.8 J/cm^2^) respectively. Irradiation times of 180 (∼112.2 J/cm^2^), 240 (149.6 J/cm^2^) and 300 (187 J/cm^2^) seconds in the absence of CFW were also tested. Each experiment was performed in triplicate.

### Disc Diffusion Assays

Disc diffusion assays were employed to visualize various interactions between CFW and drugs shown to be synergistic by MIC_80_ assays. RPMI 1640 agar plates were prepared containing 0 or 11.46 µg/ml CFW. *C. albicans* suspensions, containing approximately 1×10^6^ cfu, were spread uniformly on plates using sterile cotton-tipped applicators. Blank 6 mm sterile paper discs (Becton Dickinson) were impregnated with 10 µl volumes of a 10-fold range of drug concentrations, and after drying, were placed on inoculated plates. Plates were observed following 2 days incubation at 30°C. Experiments were performed in duplicate.

## Results and Discussion

### Inhibition by Stilbene Fluorescent Brighteners and Compounds

**Figure 1:**
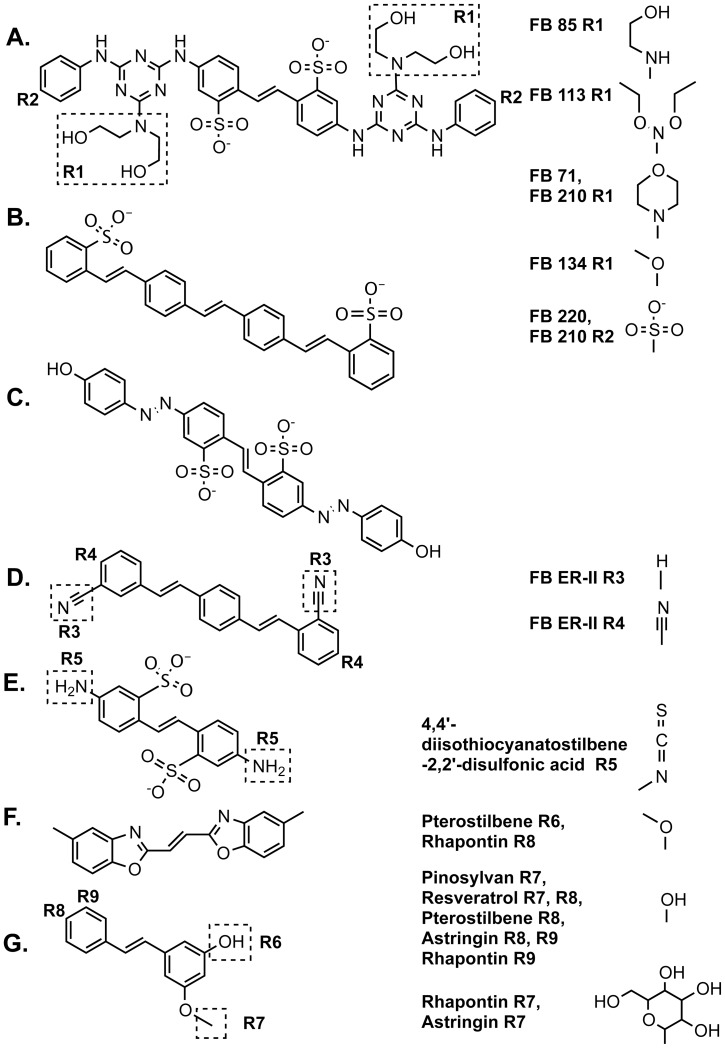
Structures of representative stilbene and non-stilbene fluorescent brighteners and stilbene phytoalexin compounds. Residues (R) at which related products differ from the representative compound are displayed. A. CFW. B. Fluorescent brightener 351. C. Brilliant yellow. D. Fluorescent brightener ER-III. E. 4,4′-diamino-2–2′-stilbenedisulfonic acid. F. Fluorescent brightener 135. G. Pinosylvan monomethyl ether.

Because CFW and the structurally similar fluorescent brightener 220 have been shown to inhibit fungal growth, we tested a panel of related stilbene and non-stilbene fluorescent brighteners (Fig. 1) for the ability to inhibit the growth of the *T. rubrum* and *C. albicans*. Consistent with previous findings, CFW was fungicidal to both *T. rubrum* (MIC_80_ 2.87 µg/ml, MFC_99_ 5.73 µg/ml) and *C. albicans* (MIC_80_ 11.46–22.92 µg/ml, MFC_95_ 22.92–45.85 µg/ml) (Table 1). A number of structurally related compounds were also fungicidal when used at similar molarities, with the most inhibitory including fluorescent brighteners 85, 113 and 71, followed by 134 (Table 1). We found no inhibition by the less structurally similar 4,4′-diamino-2–2′-stilbenedisulfonic acid, 4,4′-diisothiocyanatostilbene-2,2′-disulfonic acid disodium salt hydrate, or fluorescent brighteners ER-II or ER-III. Furthermore, while antifungal testing of the non-stilbene fluorescent brightener 135 was limited due to poor water solubility, we found no inhibition by this compound.

**Table 1:** Inhibition of *T. rubrum* ATCC MYA-4438 and *C. albicans* SC5314 by fluorescent brightener and stilbene compounds.

Compound	*T. rubrum* MIC_80_ (µg/ml)	*T. rubrum* MFC_99_ (µg/ml)	*C. albicans* MIC_80_ (µg/ml)	*C. albicans* MFC_95_ (µg/ml)	Related structure^a^	Differing residues^a^
*Fluorescent brightener (FB) stilbenes*						
CFW	2.87	5.73	11.46–22.92	22.92–45.85	A	
FB 85	2.73	5.56	10.91	21.82	A	R1
FB 113	3.00	6.01	12.01	24.02	A	R1
FB 71	2.89	5.78	11.56	>46.25	A	R1
FB 134	5.09	10.18	20.37	162.95	A	R1
FB 220	36.41	72.81	582.52	>1165.03	A	R2
FB 351 (Uvitex 2B)	28.13	56.26	28.13	>225.03	B	
Brilliant yellow	7.81	>124.91	>124.91	>124.91	C	
FB 210	112.90	>225.80	>225.80	>225.80	A	R1, R2
FB ER-II	>66.48	>66.48	>66.48	>66.48	D	R3, R4
FB ER-III	>66.48	>66.48	>66.48	>66.48	D	
4,4′-diamino-2–2′-stilbenedisulfonic acid	>74.08	>74.08	>74.08	>74.08	E	
4,4′-diisothiocyanatostilbene-2,2′-disulfonic acid	>99.70	>99.70	>99.70	>99.70	E	R5
*Non-stilbene FB*						
FB 135	>14.52	>14.52	>14.52	>14.52	F	
*Phytoalexin stilbenes*						
Pinosylvin mono methyl ether	5.66	11.31	22.63	45.26	G	
Pterostilbene	6.40	25.60	25.60	25.60	G	R6, R8
Pinosylvin	21.22	42.45	≥42.45	>42.45	G	R7
Resveratrol	>45.65	>45.65	>45.65	>45.65	G	R7, R8
Astringin	>81.20	>81.20	>81.20	>81.20	G	R7, R8, R9
Rhapontin	>84.08	>84.08	>84.08	>84.08	G	R7, R8, R9

a See Fig. 1 for structures and residues.

From our results, we have derived a structure-activity relationship for features important for fungal inhibition in the compounds tested. The compounds giving the best inhibition contained the core structure depicted in Fig. 1A. There was considerable flexibility allowed at the R1 residue, with various modifications resulting in minimal changes to fungal sensitivity, with the least tolerated change observed consisting of the substitution of R1 to a methoxy group (fluorescent brightener 134). Modification of the R2 residue from a hydrogen to a sulfate group had a greater impact on function, with at least 10-fold reduced sensitivity of strains to fluorescent brightener 220 compared with CFW, or fluorescent brightener 210 compared with fluorescent brightener 71 (the R1 groups are the same for both CFW and fluorescent brightener 220, and both fluorescent brighteners 210 and 71). Possible scenarios by which R-groups could influence fungal inhibition by fluorescent brighteners include effects on solubility, hydrogen-bonding to the substrate, conformation, or steric hindrance. While literature has concentrated on the fungal effects of fluorescent brightener binding to chitin, CFW may also bind other hexopyranose polymers in the β-conformation *in vitro* [11], as is present in β–glucans, thus any changes to the substrate specificity of fluorescent brighteners on fungal inhibition may also be exerted at the level of changes to β–glucan binding and perturbation.

Various naturally occurring phytoalexins are also stilbenoid compounds and some have been reported to have antifungal activity [44], [45], [46], [47]. Therefore, we also tested a group of structurally related stilbene phytoalexins (Fig. 1) for the ability to inhibit *T. rubrum* and *C. albicans*. In contrast to findings by Jung et al. [44], [45] who showed inhibition of a different *C. albicans* strain (TIMM 1768) by 20 µg/ml resveratrol in YPD medium, and in agreement with Weber et al. [48] who found no inhibition of three *C. albicans* (including SC5314 used in this study) or five other *Candida* species by resveratrol at ≤128 µg/ml in rich and minimal media, we found no inhibition of *C. albicans* SC5314 by resveratrol (MIC >45.65 µg/ml). Furthermore, *T. rubrum* was also not inhibited at the concentrations tested (Table 1). We also found no inhibition by astringin or rhapontin. Interestingly, both pinosylvin monomethyl ether and pterostilbene (dimethylether analog of resveratrol) inhibited *T. rubrum* and *C. albicans*, and pinosylvin inhibited *T. rubrum*, although inhibition required higher concentrations of drug than for CFW inhibition.

While the inhibitory mechanism of stilbene fluorescent brighteners is likely due to the binding of fungal chitin (and/or β–glucans**)**, affecting cell wall integrity, the antifungal target of action of stilbene phytoalexin compounds is less well understood. Disruption of drug efflux genes increased sensitivity to stilbene phytoalexins in *S. cerevisiae* and *Aspergillus nidulans*, suggesting that in contrast to CFW, the inhibitory mechanism is internal to the cell [49], [50], [51]. Furthermore, the antifungal efficacy of stilbene phytoalexins are likely correlated with their ability to be transported into cells, for example, the weak activity of resveratrol has been attributed to its hydrophilic character, making it difficult to pass through membranes [52]. The conjugated bond system of hydroxystilbenes form charge transfer complexes with affinity for proteins. These compounds have also been shown to inhibit fungal enzymes such as tyrosinases [53], affect membranes [52], and affect expression of genes required for methionine and lipid metabolism and mitochondrial function [50]. Hence, hydroxystilbene phytoalexins exert wide-ranging effects on the physiological and biochemical processes of fungi. Therefore, although the fluorescent brighteners and phytoalexins studied shown here to inhibit fungi were all stilbene compounds, the mechanisms of antifungal activity likely differ between the two groups.

### UV_365 nm_ Irradiation Reduces CFW Efficacy

Because CFW selectively binds and kills fungi at low concentrations and has a UV absorption peak of 365 nm (UVA-1), which is within the wavelength spectrum already reported to be fungicidal for *T. rubrum* (340–550 nm at 40 J/cm^2^ [54]), we tested the hypothesis that CFW and UV_365 nm_ irradiation combination treatments would be synergistic for *T. rubrum* and *C. albicans* inhibition. Irradiation alone was found to be fungicidal, with 80% inhibition of growth of *T. rubrum* at 56.2 J/cm^2^ and *C. albicans* at 149.6 J/cm^2^, 99% killing of *T. rubrum* at 74.8 J/cm^2^, and 95% killing of *C. albicans* at 149.6 J/cm^2^. Interestingly, we find that UV_365 nm_ treatment at 37.4 and 56.2 J/cm^2^ reduces both the *C. albicans* CFW MIC_80_ and MFC_95_ levels, but the same was not observed for *T. rubrum* (Table 2). However, we find that a lower level of UV_365 nm_ irradiation of 18.7 J/cm^2^ resulted in 8–32-fold increased CFW MIC_80_ and MFCs for both *T. rubrum* and *C. albicans* (Table 2). Similar results were obtained using other strains of *T. rubrum* (MR851, MR1505, MR1461 and MR827), and UV_365 nm_ irradiation at 18.7 and 37.4 J/cm^2^ also increased the *T. rubrum* MIC_80_ for fluorescent brighteners 113 and 85 to >24.02 and >21.82 µg/ml, respectively. Therefore, subinhibitory levels of UV_365 nm_ irradiation are antagonistic with CFW for both *T. rubrum* and *C. albicans*.

**Table 2:** Combination treatments with CFW and UV_365 nm_ irradiation.

UV treatment (J/cm^2^)^a^	*T. rubrum* MIC_80_ (µg/ml)	*T. rubrum* MFC_99_ (µg/ml)	*C. albicans* MIC_80_ (µg/ml)	*C. albicans* MFC_95_ (µg/ml)
0	2.87	2.87	11.46	45.85
18.7	45.85	91.70	183.40	>183.40
37.4	22.92	91.70	2.87	22.92
56.2	0	91.70	0.36	1.43

a 80% growth inhibition for UV treatment alone was 56.2 J/cm^2^ for *T. rubrum* and 149.6 J/cm^2^ for *C. albicans*. 99% and 95% fungicidal UV treatment alone was 74.8 J/cm^2^ for *T. rubrum* and 149.6 J/cm^2^ for *C. albicans*, respectively.

We first tested the hypothesis that UV_365 nm_ irradiation triggered a cellular response that resulted in reduced sensitivity to CFW, for example, by altering transcription of genes for chitin production. Although photoreceptors such as white collar 1 and 2 are absent in both species [55], it remains possible that chitin (and/or β–glucan) production could be affected by other stresses triggered by UV light, such as an oxidative stress response. Implied in this hypothesis, UV_365 nm_ irradiation of cells immediately prior to CFW addition should also reduce sensitivity to CFW. Therefore, we irradiated *C. albicans* cells in microtitre wells for 0, 18.7, or 56.2 J/cm^2^ immediately prior to CFW addition. For each UV_365 nm_ treatment, the CFW MIC_80_ remained unchanged at 11.46 µg/ml. Therefore, our results do not support the hypothesis that UV_365 nm_ induces a cellular response that antagonizes CFW action.

To test the hypothesis that UV_365 nm_ irradiation antagonism of CFW treatment is due to photoinactivation of the CFW, we irradiated CFW solutions at 0, 18.7, or 56.2 J/cm^2^ immediately prior to its addition to *C. albicans* cells. CFW that had been irradiated for 0, 18.7, or 56.2 J/cm^2^ resulted in *C. albicans* MIC_80 _s of 11.46, 22.92 and 45.85 µg/ml, respectively, indicating that UV_365 nm_ treatment was indeed inactivating the CFW. Inactivation is likely not due to heat inactivation caused by heat emitted by the diode, as CFW MIC_80 _s were unchanged from CFW treatment alone when wells containing *C. albicans*, RPMI 1640 medium and CFW, covered by foil (which blocks light yet should still allow heat transfer), were irradiated. Furthermore, no inhibition of *C. albicans* was observed even up to 5 min irradiation (187 J/cm^2^) of covered wells containing no CFW, and thus heat from UV_365 nm_ irradiation treatment was also not affecting *C. albicans* growth in the absence of CFW. Therefore, reduced sensitivity of *C. albicans* and *T. rubrum* to CFW in the presence of UV_365 nm_ irradiation is likely due to photoinactivation of CFW. Stilbene compounds are known to undergo photoisomerization, photodimerization, and photocyclization reactions in the presence of UV due to the nature of their conjugated bond system [56], [57], thus any photoinactivation of CFW is not unexpected.

Therefore, while CFW and UV_365 nm_ irradiation are individually fungicidal to both *T. rubrum* and *C. albicans*, the combination treatment was antagonistic, and not a viable treatment option. Similarly, rather than increasing any inhibitory effect, subinhibitory concentrations of stilbene fluorescent brighteners in combination with UV_254 nm_ increased spore germination in various fungi [16], [58], and protected cells from UV_254 nm_ damage [16], although we did not observe any CFW-mediated protection from UV_365 nm_ inhibition in our studies. Possible photoinactivation of CFW by UV light raises the concern that efficacy of topical CFW treatment could be reduced by exposure to sunlight. However, given that topical application concentrations of CFW would be higher than the MIC of the infecting fungus, and UV irradiation in these experiments is at substantially higher levels than that present in natural sunlight, photoinactivation effects would be negligible, and could be avoided completely by covering the treatment area.

### Drug Interactions with CFW

**Figure 2:**
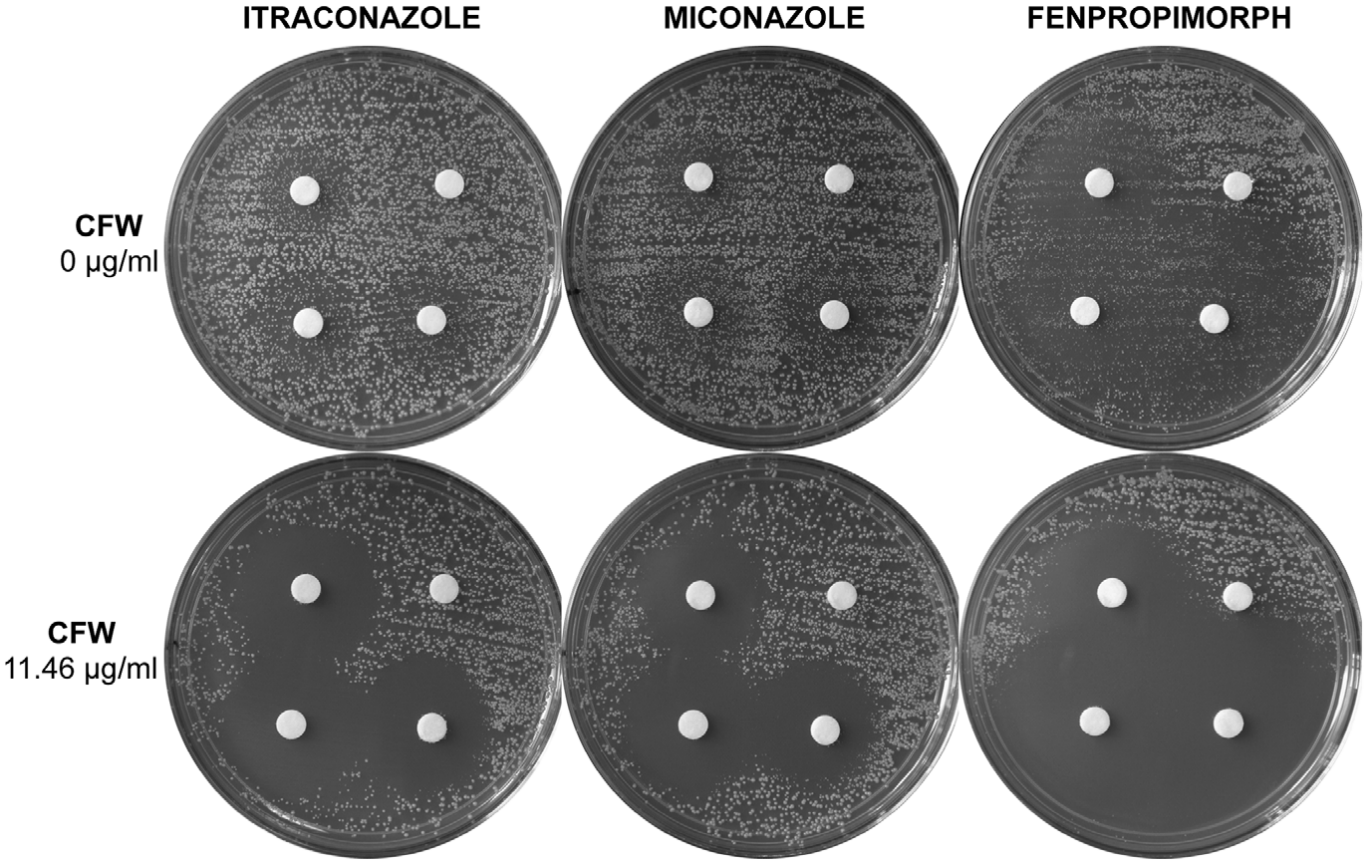
Disc diffusion assays showing enhanced *C. albicans* inhibition when treated with CFW and ergosterol biosynthesis inhibitors. Starting at the bottom right disc and moving clockwise, the amounts of each drug added per disc included 14.11, 3.52, 1.41 and 0 ng of itraconazole, 8.32, 2.08, 0.83, and 0 ng of miconazole, and 6.07, 1.52, 0.61, and 0 ng of fenpropimorph. Drugs were diluted in DMSO, and DMSO comprised the no-drug control.

Combination drug treatment regimes may provide a broader spectrum of action and reduce the emergence of drug resistance. Therefore, using checkerboard assays, we investigated whether CFW had synergistic inhibitory effects with other inhibitors of *T. rubrum* and/or *C. albicans* with differing cellular targets. All MIC_80_ results are listed in Table 3. Disc diffusion assays were also performed for *C. albicans* with drugs that had a synergistic interaction with CFW (Fig. 2).

**Table 3:** Tabele 3. *T. rubrum* ATCC MYA-4438 and *C. albicans* SC5314 combination drug treatments with CFW.

Drug	*T. rubrum*			*C. albicans*		
	Individual MIC_80_(µg/ml)(CFW/drug)	Combined MIC_80_(µg/ml)(CFW/drug)	FIC	Individual MIC_80_(µg/ml)(CFW^a^/drug)	Combined MIC_80_(µg/ml)(CFW/drug)	FIC
*Azoles*						
Clotrimazole	2.87/0.034	1.43/0.017	1	11.46/0.022	1.43/0.011	0.63
Itraconazole	2.87/0.110	1.43/0.028	0.75	11.46/0.022	5.73/0.003	0.63
Miconazole	2.87/0.150	2.87/0.150	2	22.92/2.40	5.73/0.019	0.26
Thioconazole	2.87/0.060	2.87/0.060	2	5.73/0.006	0.72/0.003	0.63
Voriconazole	2.87/0.014	2.87/0.014	2	11.46/0.011	5.73/0.003	0.75
*Allylamines*						
Butenafine HCl	2.87/1.77	1.43/0.88	1	22.92/>17.70	22.92/>17.70	NA^a^
Terbinafine HCl	2.87/4.10	1.43/2.05	1	22.92/>131.16	11.46/1.02	NA^b^
*Other*						
Griseofulvin	2.87/0.55	1.43/0.28	1	22.92/>17.74	22.92/>17.74	NA^b^
Manumycin A	2.87/10.32	2.87/10.32	2	22.92/22.03	5.73/11.01	0.75
Fenpropimorph	2.87/0.237	2.87/0.237	2	22.92/15.17	2.87/0.030	0.13
Rapamycin	2.87/>45.71	0.72/1.43	NA^b^	11.46/0.09	5.73/0.023	0.75
Nikkomycin Z	2.87/>49.54	2.87/>49.54	NA^b^	11.46/>24.77	>45.85/6.19	NA^b^

a Some experiment-to-experiment differences in CFW MIC_80 _s for *C. albicans* were observed, such as for experiments where the MIC_80 _s were read after 24 h instead of 48 h.

b Not applicable. When the MIC_80_ for any drug was higher than the highest concentration tested, the FIC could not be determined.

Nikkomycin Z inhibits chitin synthase, reducing levels of the CFW target, chitin [59]. Conversely, treatment with CFW and other fluorescent brighteners increases chitin production in *S. cerevisiae* and *C. albicans* [19], [60], [61]. Therefore, we predicted that nikkomycin Z would be antagonistic with CFW, as has been observed previously with nikkomycin Z and CFW and related fluorescent brighteners in *S. cerevisiae* [59], [62]. For *T. rubrum*, nikkomycin Z had no inhibitory effect alone and did not affect sensitivity to CFW. However, consistent with previous results, increasing amounts of nikkomycin Z antagonized *C. albicans* sensitivity to CFW, increasing the CFW MIC_80_ to >45.85 µg/ml with nikkomycin Z concentrations at, or exceeding, 6.19 µg/ml.

Manumycin A inhibits farnesyltransferases, a target not yet exploited for fungicides. We found that manumycin A inhibited both *T. rubrum* (MIC_80_ 10.32 µg/ml) and *C. albicans* (MIC_80_ 22.02 µg/ml) and was fungicidal to *T. rubrum* (MFC_99_ 20.64 µg/ml; MFCs were not tested for *C. albicans*). The FIC values for treatment with CFW and manumycin A were 2 for *T. rubrum* and 0.75 for *C. albicans*; thus the drug interaction in both species was indifferent.

The antibiotic griseofulvin binds tubulin, interfering with microtuble function and inhibiting mitosis, and is administered orally for dermatophyte infections. Although *C. albicans* was not inhibited by griseofulvin at the concentrations used (≤22.92 µg/ml), *T. rubrum* was sensitive with an MIC_80_ of 0.55 µg/ml, comparable with previous results (0.125–4 µg/ml, incubation at 28°C, 7 days [63]), and the interaction of griseofulvin in combination with CFW was indifferent (FIC 1).

The immunosuppressive drug rapamycin inhibits the TOR pathway via binding to FKBP12 [64], and is synergistic with other antifungals [65], [66]. Although the use of an immunosuppressant may not be recommended as an orally administered antifungal treatment since many patients are already immunocompromised, topical applications may have utility. While rapamycin did not inhibit *T. rubrum* at the concentrations used (≤45.71 µg/ml), we observed a positive interaction with CFW treatment, with a four-fold reduced CFW MIC_80_ at rapamycin concentrations ≥1.43 µg/ml. Rapamycin treatment reduced the CFW MIC_80_ in *C. albicans*, although the FIC observed of 0.75 is defined as indifferent.

The azole drugs (clotrimazole, itraconazole, miconazole, thioconazole and voriconazole) inhibit lanosterol 14α-demethylase, the allylamine drugs (butenafine HCl and terbinafine HCl) inhibit squalene epoxidase, and the morpholine fenpropimorph inhibits both sterol C_8_–C_7_ isomerase and C-14-reductase; all of these enzymes are required for the synthesis of the membrane component ergosterol [26]. For *T. rubrum*, sensitivities to ergosterol synthesis inhibitors in the absence of CFW were comparable to previously published result ranges, for example the voriconazole MIC_80_ of 0.014 µg/ml, miconazole MIC_80_ of 0.150 µg/ml, and clotrimazole MIC_80_ of 0.034 µg/ml, were within the published ranges of 0.008–0.06 µg/ml (for incubation at 35°C) [28], 0.031–4 µg/ml, and 0.031–0.5 µg/ml (for incubation at 28°C, 7 days) [63], respectively. We found no synergistic interaction for CFW with any of the ergosterol biosynthesis inhibitors. Butenafine HCl and terbinafine HCl did not inhibit *C. albicans* at any of the concentration tested. The *C. albicans* MIC_80 _s for other ergosterol biosynthesis-inhibiting drugs were also within published ranges for microdilution assays incubated at 35°C, for example the voriconazole MIC_80_ of 0.011 µg/ml and itraconazole MIC_80_ of 0.022 µg/ml, were within the range of 0.007–1 µg/ml [31], 0.008–2 µg/ml [67], respectively. Clotrimazole, itraconazole, thioconazole, and voriconazole all reduced the CFW MIC_80_ for *C. albicans*, however, FIC values were all within the range considered indifferent. Despite the interaction between these drugs and CFW being designated as indifferent, we observed increased halos of inhibition by a subset of these compounds (itraconazole, Fig. 2; and clotrimazole, data not shown) in the presence of CFW. Interestingly, miconazole and fenpropimorph were both synergistic in combination with CFW (FIC 0.26 and 0.13, respectively). Disc diffusion assays also indicated enhanced inhibition of *C. albicans* by combination treatments of CFW and miconazole, and fenpropimorph, than either drug individually. Not only did CFW result in wider halos of inhibition for each drug, but also the elimination of the partial growth of colonies within halos observed for each drug in the absence of CFW (Fig. 2).

Chitin synthesis is under tight spatial and temporal regulation, and cell wall perturbations caused by CFW treatment trigger signaling cascades, such as the HOG, PKC and calcineurin pathways, to respond to cell wall damage and make compensatory changes in *S. cerevisiae* and *C. albicans* [60], [68]. Therefore, drugs that either affect chitin levels or signaling cascades that respond to CFW-mediated perturbations could have interactions with CFW. For example, in addition to the nikkomycin Z/fluorescent brightener antagonism discussed previously [62], pretreatment with CFW ameliorates *C. albicans* sensitivity to echinocandin by stimulation of chitin synthesis [69]. A mechanism for the positive interaction between ergosterol biosynthesis inhibition and CFW in *C. albicans* is unclear, although synergism between inhibition of chitin synthesis (nikkomycin Z) and azoles has also been demonstrated in this species [70], [71], [72]. The synergism between nikkomycin Z and azoles has been attributed to ergosterol biosynthesis inhibitors perturbing the synthesis, transport to the membrane, or stability of membrane-located chitin synthase [71], [73], [74]. However, given our results that the chitin synthase inhibitor nikkomycin Z is antagonistic rather than synergistic with CFW, any inhibition of chitin production by ergosterol inhibitors would not explain the synergistic interaction with CFW observed in *C. albicans*. Others have reported that reduced ergosterol production instead causes enhanced chitin synthesis and irregular distribution, which could be detrimental to cells already compromised in chitin assembly and cell wall integrity by CFW treatment, and indeed, results in sensitivity to CFW [75]. To test whether reduced cell wall integrity contributed to the synergistic effect of CFW and ergosterol inhibitors, we determined the effect of the osmotic stabilizer sorbitol on CFW and fenpropimorph combination treatment of *C. albicans* using checkerboard assays. The presence of sorbitol (1 M) increased by >2-fold the individual CFW MIC_80_, and did not alter the individual fenpropimorph MIC_80_. Consistent with the reduced cell wall integrity hypothesis, sorbitol addition partially ameliorated inhibition by fenpropimorph in the presence of CFW, with a 4-fold increased CFW concentration required to inhibit growth in combination with subinhibitory concentrations of fenpropimorph for which a synergistic interaction was previously observed. In contrast to *C. albicans*, the lack of synergistic interaction between ergosterol biosynthesis inhibitors and CFW in *T. rubrum* may be due to different mechanisms of regulation of chitin synthesis between the divergent species, and the possibility that in addition to chitin binding, CFW may also effect *T. rubrum* growth by binding β–glucan present in the outermost cell layer of this species.

### Conclusions

In an endeavor to extend the compendium of effective treatments for superficial mycoses, we have identified various CFW-related stilbene fluorescent brighteners and stilbene phytoalexins with fungicidal activity against a representative strain of both *T. rubrum* and *C. albicans*. Furthermore, we found several synergistic interactions with CFW and clinically relevant ergosterol synthesis-inhibiting antifungal drugs for *C. albicans*, offering potential for combination treatments that increase the spectrum of antifungal activity, while reducing the chance of drug resistance arising. The efficacy of particular CFW and related compound treatments and combinations may be somewhat environment-, strain-, species-, and cell morphology-specific due to the differences in mechanisms controlling chitin synthesis and differences in cell wall composition. Therefore, in the future it would be interesting to test our findings using more *C. albicans* and *T. rubrum* isolates, different dermatophyte species, to compare hyphal and arthrospore susceptibilities, and in different environmental conditions such as high and low temperatures. Nonetheless, the importance of chitin in the cell walls of spores, hyphae and yeast cells of fungi suggest that targeting this unexploited antifungal target with CFW and related compounds will have widespread application against all cell morphologies from evolutionarily diverged fungi.

## Supporting Information

Table S1 **Drugs used in this study.**
(DOCX)
