# Supplementary material for: Calcofluor White Combination Antifungal Treatments for Trichophyton rubrum and Candida albicans
Source: PLoS One. 2012 Jul 6;7(7):e39405. doi: 10.1371/journal.pone.0039405 (PMC3391284; doi:10.1371/journal.pone.0039405)
Supplement: Table S1 — Drugs used in this study. (DOCX) [file pone.0039405.s001.docx]

**Table S1. Drugs used in this study.**

| **Drug** | **CAS** | **Company** | **Diluent** | **Concentration range (µg/ml)^a^** |
| --- | --- | --- | --- | --- |
| Calcofluor white (CFW) | 4404-43-7 | Sigma | Water | 0.18-183 |
| Fluorescent brightener (FB) 71 | 16090-02-1 | AK Scientific | DMSO | 0.36-369.96 |
| FB 85 | 12224-06-5 | AK Scientific | Water | 0.68-698.27 |
| FB 113 | 12768-92-2 | AK Scientific | Water | 0.19-192.19 |
| FB 134 | 3426-43-5 | AK Scientific | Water | 0.16-162.95 |
| FB 135 | 1041-00-5 | AK Scientific | Ethanol | 0.01-14.52 |
| FB 210 | 28950-61-0 | AK Scientific | Water | 0.23-225.80 |
| FB 220 | 16470-24-9 | Waterstone Technology, LLC | Water | 1.14-1165.03 |
| FB 351 (Uvitex 2B) | 27344-41-8 | Polysciences, Inc | Water | 0.22-225.03 |
| FB ER-II | 13001-38-2 | AK Scientific | DMSO | 0.07-66.48 |
| FB ER-III | 79026-03-2 | AK Scientific | DMSO | 0.07-66.48 |
| Brilliant yellow | 3051-11-4 | Sigma | Water | 0.12-124.91 |
| Pinosylvin mono methyl ether | 35302-70-6 | Sequoia Research Products Ltd | DMSO | 0.05-45.27 |
| Pterostilbene | 537-42-8 | Sequoia Research Products Ltd | DMSO | 0.05-51.20 |
| Pinosylvin | 22139-77-1 | Sequoia Research Products Ltd | DMSO | 0.04-42.45 |
| Resveratrol | 501-36-0 | Sigma | Ethanol | 0.05-45.65 |
| Astringin | 29884-49-9 | Sequoia Research Products Ltd | Water | 0.81-81.20 |
| Rhapontin | 155-58-8 | Sinova | DMSO | 0.08-84.08 |
| 4,4’-diamino-2-2’-stilbenedisulfonic acid | 81-11-8 | Sigma | 1 M NaOH | 0.07-74.08 |
| 4,4'-diisothiocyanatostilbene-2,2'-disulfonic acid disodium salt hydrate | 207233-90-7 | Sigma | Water | 0.10-99.70 |
| Terbinafine HCl | 78628-80-5 | Sigma, AK Scientific | DMSO/Ethanol | 0.13-131.16 (Ca)  0.03-32.79 (Tr) |
| Butenafine HCl | 101827-46-7 | Sigma | DMSO | 0.02-17.70 (Ca)  0.01-7.08 (Tr) |
| Voriconazole | 137234-62-9 | Sigma | DMSO | 0.0007-0.70 (Ca)  0.007-6.99 (Tr) |
| Itraconazole | 84625-61-6 | Sigma | DMSO | 0.001-1.41 (Ca)  0.007-7.06 (Tr) |
| Thioconazole | 65899-73-2 | Sigma | Ethanol | 0.0008-0.78 (Ca)  0.008-7.75 (Tr) |
| Clotrimazole | 23593-75-1 | Sigma | DMSO | 0.0007-0.69 (Ca)  0.017-17.24 (Tr) |
| Miconazole | 22832-87-7 | Sigma | DMSO | 0.010-9.58 (Ca)  0.005-4.79 (Tr) |
| Manumycin A | 52665-74-4 | Alexis Biochemicals | DMSO | 0.021-22.03 (Ca)  0.083-82.60 (Tr) |
| Fenpropimorph | 67564-91-4 | Crescent Chemicals | DMSO | 0.030-30.35 |
| Rapamycin | 53123-88-9 | LC Laboratories | Ethanol or DMSO | 0.0009-0.091 (Ca)  0.045-45.71 (Tr) |
| Griseofulvin | 126-07-8 | Sigma | DMSO | 0.02-17.74 |
| Nikkomycin Z | 59456-70-1 | Sigma | Water | 0.02-24.77 (Ca)  0.05-49.54 (Tr) |

^a^ When concentration ranges tested differed between *C. albicans* (Ca) and *T. rubrum* (Tr), both ranges are listed.
